# Cytotoxicity of ascorbate, lipoic acid, and other antioxidants in hollow fibre in vitro tumours

**DOI:** 10.1054/bjoc.2001.1814

**Published:** 2001-06

**Authors:** J J Casciari, N H Riordan, T L Schmidt, X L Meng, J A Jackson, H D Riordan

**Affiliations:** Bio-Communications Research Institute, Center for the Improvement of Human Functioning International, 3100 North Hillside Avenue, Wichita, KS 67219; Department of Medical Technology, Wichita State University, Wichita, KS 67260

**Keywords:** ascorbic acid (vitamin C), thioctic acid (lipoic acid), vitamin K, tumour cells, cultured, dose–responseelationship, drug

## Abstract

Vitamin C (ascorbate) is toxic to tumour cells, and has been suggested as an adjuvant cancer treatment. Our goal was to determine if ascorbate, in combination with other antioxidants, could kill cells in the SW620 hollow fibre in vitro solid tumour model at clinically achievable concentrations. Ascorbate anti-cancer efficacy, alone or in combination with lipoic acid, vitamin K _3_, phenyl ascorbate, or doxorubicin, was assessed using annexin V staining and standard survival assays. 2-day treatments with 10 mM ascorbate increased the percentage of apoptotic cells in SW620 hollow fibre tumours. Lipoic acid synergistically enhanced ascorbate cytotoxicity, reducing the 2-day LC _50_ in hollow fibre tumours from 34 mM to 4 mM. Lipoic acid, unlike ascorbate, was equally effective against proliferating and non-proliferating cells. Ascorbate levels in human blood plasma were measured during and after intravenous ascorbate infusions. Infusions of 60 g produced peak plasma concentrations exceeding 20 mM with an area under the curve (24 h) of 76 mM h. Thus, tumoricidal concentrations may be achievable in vivo. Ascorbate efficacy was enhanced in an additive fashion by phenyl ascorbate or vitamin K _3_. The effect of ascorbate on doxorubicin efficacy was concentration dependent; low doses were protective while high doses increased cell killing. © 2001 Cancer Research Campaign http://www.bjcancer.com


British Journal of Cancer (2001) 84(11), 1544–1550
© 2001 Cancer Research Campaign
doi: 10.1054/ bjoc.2001.1814, available online at http://www.idealibrary.com on IDEAL®

http://www.bjcancer.com

### Cytotoxicity of ascorbate, lipoic acid, and other antioxidants in hollow fibre in vitro tumours

**JJ Casciari[1], NH Riordan[1,*], TL Schmidt[1], XL Meng[1], JA Jackson[2] and HD Riordan[1]**

[1]Bio-Communications Research Institute, Center for the Improvement of Human Functioning International, 3100 North Hillside Avenue, Wichita, KS 67219;
[2]Department of Medical Technology, Wichita State University, Wichita, KS 67260

**Summary** Vitamin C (ascorbate) is toxic to tumour cells, and has been suggested as an adjuvant cancer treatment. Our goal was to determine if ascorbate, in combination with other antioxidants, could kill cells in the SW620 hollow fibre in vitro solid tumour model at clinically achievable concentrations. Ascorbate anti-cancer efficacy, alone or in combination with lipoic acid, vitamin $K_3$, phenyl ascorbate, or doxorubicin, was assessed using annexin V staining and standard survival assays. 2-day treatments with 10 mM ascorbate increased the percentage of apoptotic cells in SW620 hollow fibre tumours. Lipoic acid synergistically enhanced ascorbate cytotoxicity, reducing the 2-day $LC_{50}$ in hollow fibre tumours from 34 mM to 4 mM. Lipoic acid, unlike ascorbate, was equally effective against proliferating and non-proliferating cells. Ascorbate levels in human blood plasma were measured during and after intravenous ascorbate infusions. Infusions of 60 g produced peak plasma concentrations exceeding 20 mM with an area under the curve (24 h) of 76 mM h. Thus, tumoricidal concentrations may be achievable in vivo. Ascorbate efficacy was enhanced in an additive fashion by phenyl ascorbate or vitamin $K_3$. The effect of ascorbate on doxorubicin efficacy was concentration dependent; low doses were protective while high doses increased cell killing. © 2001 Cancer Research Campaign http://www.bjcancer.com

**Keywords**: ascorbic acid (vitamin C); thioctic acid (lipoic acid); vitamin K; tumour cells, cultured; dose–response relationship, drug

Vitamin C (ascorbic acid, ascorbate) is a major water soluble antioxidant with a variety of biological functions. It plays roles in collagen and carnitine synthesis and may be important in maintaining proper immune cell function (Goldschmidt, 1991; Penn et al, 1991). In rodents, vitamin C protects normal tissues from the oxidative damage associated with chemotherapy and radiation (Fujita et al, 1982; Okunieff and Suit, 1987). These and other observations have created interest in using vitamin C as an adjuvant treatment for cancer (Henson et al, 1991).

While vitamin C commonly functions as an antioxidant, it can also act as a pro-oxidant by converting free radicals into hydrogen peroxide, a molecule that can damage cell membranes and DNA if not neutralized by the cellular enzyme catalase (Koch and Biaglow, 1978). At sufficient concentrations, vitamin C can produce cytotoxic levels of hydrogen peroxide. Tumour cells are often catalase deficient and therefore more sensitive than normal to hydrogen peroxide (Benade et al, 1969). Vitamin C accumulates in solid tumours at concentrations higher than those in surrounding normal tissue (Langemann et al, 1989; Agus et al, 1999). This has raised concerns that vitamin C may provide tumours with antioxidant protection from traditional therapeutic modalities (Raloff, 2000). However, vitamin C may be useful as an anti-cancer agent if cytotoxic ascorbate concentrations can be achieved in tumours (Riordan et al, 1995).

Experiments in rodents suggest that ascorbate administration increases host survival times and inhibit tumour growth (Varga and Airoldi, 1983; Tsao et al, 1988). Clinical trials with vitamin C have yielded mixed results. In 2 Scottish studies, terminal cancer patients given intravenous vitamin C (10 g $day^{-1}$) showed longer survival times than historical controls (Cameron and Campbell, 1974; Cameron and Pauling, 1976). A Japanese study yielded similar results (Murata et al, 1982), but 2 double-blind studies at the Mayo clinic using oral vitamin C (10 g $day^{-1}$) showed no benefit (Creagan et al, 1979; Moertel et al, 1985). Oral vitamin C supplementation is unlikely to produce plasma ascorbate levels sufficient to kill tumour cells directly (Riordan et al, 1995). Intravenous vitamin C at higher doses has been effective in individual cases (Riordan et al, 1990, 1998; Jackson et al, 1995).

We tested the anti-cancer efficacy of vitamin C and other antioxidants (vitamin $K_3$, lipoic acid, phenyl ascorbate) using the SW620 hollow fibre solid tumour (HFST) model, a heterogeneous in vitro model that shares important traits with solid tumour microregions (Casciari et al, 1994). We also examined the effect of vitamin C on doxorubicin efficacy in HFST. Finally, we measured ascorbate concentrations in human plasma during and intravenous infusions to determine if cytotoxic levels were clinically achievable.

### MATERIALS AND METHODS

### Cell culture

SW620 human colon carcinoma cells (ATCC, Rockville, MD) were grown in RPMI medium (Irvine Scientific, Santa Anna, CA) supplemented with $10^5$ U $l^{-1}$ penicillin, $10^5$ µg $l^{-1}$ streptomycin, 10% fetal bovine serum (Sigma Chemical Co, St Louis, MO) and

*Received 11 August 2000*
*Revised 23 January 2001*
*Accepted 9 February 2001*

*Correspondence to:* JJ Casiari

*Aidan Incorporated, 621 South 48th Street, Suite 111, Tempe, AZ 85281

2 mM glutamine (Irvine Scientific, Santa Anna, CA). The cells were incubated at 37˚C with a gas phase of 5% $CO_2$ in air at 100% relative humidity. Cells were passaged weekly, and replaced with fresh cells from liquid nitrogen storage every 6 months. Cells were harvested from monolayer cultures using a standard trypsin and EDTA solution (Irvine Scientific, Santa Anna, CA). The following additional cell lines, all from ATCC (Rockville, MD), were used in monolayer dose–response experiments: CCD-18Co (human colon fibroblast), CCD-25Sk (human skin fibroblast), CCD-18Lu (human lung fibroblast), SK-Mel (human melanoma), Mia PaCa (human pancreatic carcinoma), and MCF-7 (human breast carcinoma). These cells were maintained in Dulbecco's Modified Eagle Medium (Irvine Scientific, Santa Anna, CA) supplemented with serum, glutamine and antibiotics as described above.

### Reagents

Sodium ascorbate, lipoic acid (6,8-thioctic acid), doxorubicin, vitamin $K_3$ (menadione sodium bisulfate) were obtained from Sigma Chemical Co (St Louis, MO). The sodium salt of lipoic acid was prepared by mixing lipoic acid (0.1 M) with sodium bicarbonate (0.1 M) in de-ionized water. The solution was then lyophilized to recover a yellow-green powder. Analysis using an UV spectrophotometer indicated that the sodium salt had the same maximum absorbance, 330 nm, as lipoic acid. Phenyl ascorbate was kindly provided by Dr Kandatege Wimalasena (Wichita State University, Wichita, KS). In combination studies, concentration ratios were selected so that the concentration of the second agent would be sub-lethal when that of ascorbate was in the effective range. The following mass ratios were used: a 40:1 ratio of ascorbate to vitamin $K_3$; a 100:1 ratio of ascorbate to lipoic acid; a 10:1 ratio of ascorbate to the sodium salt of lipoic acid; and a 10 000:1 ratio of ascorbate to doxorubicin. In the case of lipoic acid, the ratio was restricted by poor solubility.

### Cell growth inside hollow fibres

SW620 HFST were grown as described previously (Casciari et al, 1994). Briefly, 500 µm internal diameter PVDF hollow fibres with a $5 \times 10^5$ Dalton molecular mass cutoff (Spectrum Laboratories, Laguna Hills, CA) were soaked in 70% ethanol for one week and then soaked in tissue culture medium with serum for at least one day. These fibres were then injected with a suspension of $10^7$ SW620 cells $ml^{-1}$ and heat sealed at roughly 1 cm intervals. After 3 days of incubation, the growth medium was replenished and the dishes were transferred to a Rotomix 50800 orbital shaker rotating at 120 rpm (Thermolyne, Duboque, IA). Medium was replenished every other day thereafter. SW620 HSFT were used in dose–response experiments after either 2 days (sparse) or 10 days (solid).

### Dose–response experiments

Dose–response experiments using SW620 HFST were conducted as described previously (Casciari et al, 1994). Individual fibres were transferred to 12-well plates (Becton Dickinson, Franklin Lanes, NJ) containing 1 ml growth medium. Reagents were added at various concentrations and the fibres were incubated for 2 days. Cells were then extruded from the fibres, dispersed using a trypsin and EDTA solution, counted (Model ZM, Coulter Corporation, Miami, FL), and seeded in 96-well plates using a concentration of 5000 cells per well. After 6 days, the cells were fixed and stained with the protein marker sulforhodamine B (SRB) (Sigma Chemical Co, St Louis, MO). The stain was then dissolved in 10 mM Tris and the absorbance at 565 nm was determined using an Emax microplate reader (Molecular Devices, Sunnyvale, CA). To determine $LC_{50}$, the concentration at which the surviving fraction is 50%, dose–response data from replicate experiments were pooled and fit to a sigmoidal curve as described previously (Casciari et al, 1994). All data fits were carried out with the program KaleidaGraph (Synergy Software, Reading, PA), which also provided standard errors estimates for $LC_{50}$. To assess synergy in combination therapy, the ascorbate $LC_{50}$ of the combination treatment was compared to that of a hypothetical 'additive' survival curve. The 'additive' curve was generated by multiplying surviving fractions for ascorbate treatment alone to those, at appropriate concentrations, for treatment with the other agent. In experiments with cell monolayers, cells were seeded in 96-well plates at concentrations of either $6 \times 10^3$ cells per well (exponential) or $24 \times 10^3$ cells per well (confluent) in 100 µl growth medium. Various concentrations of ascorbate or lipoic acid were added to the wells and the cells were incubated for 3 days. At this time, cells were fixed and the cell number was determined using the SRB assay. $LC_{50}$ values were determined as described above.

### Apoptosis assays

The extent of apoptosis in ascorbate-treated and -untreated HFST was determined by dual staining with FITC conjugated annexin V (AV) and propidium iodide (PI). Annexin V has a natural affinity for phospholipid phosphatidylserine, a membrane molecule that trans-locates from the intracellular side of the membrane to the extracellular side soon after the initiation of apoptosis. HFST were treated for 2 days with various concentrations of ascorbate as described above. Cells were then collected from the hollow fibres. Some were seeded in 96-well plates to measure surviving fraction with the SRB assay, while the remainder were stained using the ApoAlert™ Annexin V FITC Apoptosis Kit (Clonotech Laboratories, Palo Alto, CA) for analysis using an EPICS XL-MCL flow cytometer (Coulter Corporation, Miami, FL). The excitation wavelength was 488 nm and the detection wavelengths were 525 nm and 620 nm for AV and PI, respectively. Cells staining negative for both markers were considered viable, since AV and PI are membrane impermeable, while cells staining positive for annexin V only were considered apoptotic and cells staining positive for both markers were considered necrotic. Percentages of viable, apoptotic, and necrotic cells were determined as a function of ascorbate concentration.

### Plasma vitamin C concentration measurements

To analyse vitamin C pharmacokinetics during and after intravenous infusion, we gave a series of infusions to a 72-year-old male who was in excellent physical condition except for slowly progressing, non-metastatic carcinoma of the prostate. The protocol for intravenous vitamin C infusion has been described elsewhere (Jackson et al, 1995). Briefly, the desired amount of a concentrated (500 mg $ml^{-1}$) medical grade buffered vitamin C solution (Steris Laboratories, Phoenix, AZ) was added to Lactated Ringer's solution (250 to 500 ml) and administered by a slow intravenous 'drip'. Venous blood samples collected before and up to 24 hours after infusion were centrifuged to isolate plasma,

© 2001 Cancer Research Campaign 

which was then stabilized using 3% metaphosphoric acid (4.5 ml acid to 3 ml plasma). Plasma ascorbate concentrations were determined by reduction of 2,6-dichlorophenolindophenol (Henry et al, 1974). Infusions were given over a 2-month period, with ascorbate doses of 15 g to 65 g and infusion times of 45 to 160 minutes. Plasma ascorbate concentration profiles were fit to a standard 2-compartment pharmacokinetic model (Collins, 1996). The pharmacokinetic equations, in terms of mass action kinetics, are given below:

$$\frac{dQ_P}{dt} = G(t) + K_2Q_T - (K_1 + K_E)\,Q_P \qquad (1)$$

$$\frac{dQ_T}{dt} = K_1Q_P - K_2Q_T \qquad (2)$$

Where $Q_T$ represents the amount of vitamin C in the tissue compartment and $Q_P$ is the product of the plasma ascorbate concentration and the plasma volume, which was fixed at 30 dl. The 3 adjustable volume normalized transport parameters are: $K_1$ (plasma to tissue), $K_2$ (tissue to plasma), and $K_E$ (excretion). After fitting the pharmacokinetic data to these equations and determining these rate constants, the model was used to compute the area under the curve, an expression of ascorbate exposure, over a one-day period. All calculations were performed using Excel (Microsoft Corporation, Seattle, WA).

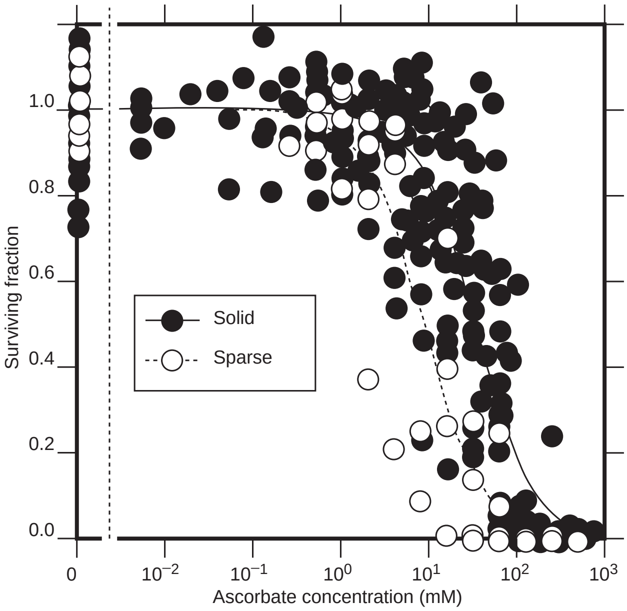

**Figure 1** Dose–response curves for SW620 cells grown 10 days as solid hollow fibre tumours (solid circles) or 2 days as sparse cell populations (open circles) inside hollow fibres. Cells were treated for 2 days with vitamin C. $LC_{50}$ values of 8.4 ± 1.5 mM and 33.7 ± 2.1 mM were obtained for sparse cell populations and HFST, respectively

### RESULTS

Apoptosis in SW620 HFST (Casciari et al, 1994) was confirmed in this study by dual staining with propidium iodide and annexin V. Cells grouped into 3 clusters; low PI and annexin V staining cells with high forward scatter and low granularity; low PI and high annexin V staining cells with low forward scatter and high granularity; and cells with high levels of both PI and annexin V. Table 1 shows the percentages of viable, apoptotic and necrotic cells from SW620 HFST treated with various concentrations of ascorbate. The viable cell fractions decreased while the apoptotic and necrotic cell fractions increased with increasing ascorbate concentration. This trend was consistent with the effects of ascorbate on surviving fraction. Ascorbate concentrations on the order of 10 mM (~200 mg $dl^{-1}$) were required to kill a significant percentage of the tumour cells. We conducted a dose–response experiment with sodium chloride to determine what osmotic and sodium levels SW620 HFST could tolerate. Sodium chloride concentrations of 180 mM were necessary to reduce HFST cell survival over a 2-day period. Thus, ascorbate toxicity at 10 mM was not due to hyperosmotic effects. Ascorbate dose–response curves collected over a 3-year period for SW620 HFST and sparse SW620 cell populations are shown in Figure 1. The $LC_{50}$ for SW620 HFST was roughly 4 times higher than that for sparse SW620 cells, a population comparable to exponentially growing cell monolayers. We expect longer exposure times to decrease survival, since surviving fraction was related to the product of concentration and time in treatments from one hour to 48 hours (data not shown).

To determine if cytotoxic ascorbate concentrations (on the order of 10 mM) could be obtained in vivo, we measured plasma concentrations with time in a volunteer during 9 intravenous ascorbate infusions and fit data from each infusion with a 2-compartment model. The average pharmacokinetic parameter values from 9 experiments were as follows: $K_E$ = 0.025 ± 0.002 $min^{-1}$, $K_1$ = 0.35 ± 0.26 $min^{-1}$ and $K_2$ = 0.10 ± 0.07 $min^{-1}$. The excretion constant ($K_E$) was remarkably reproducible and, while $K_1$ and $K_2$ had higher standard deviations, the ratio $K_2/K_1$ was also quite reproducible (0.29 ± 0.03). Plasma vitamin C concentrations for 60 and 30 gram infusions, each over 80 minutes, are shown in Figure 2. With 60 grams infused, plasma concentrations reached a maximum value of 21.8 mM (432 mg $dl^{-1}$) and then slowly decreased as the vitamin was cleared or absorbed. The resulting area under the curve, an expression of total ascorbate exposure, was 76 mM h over a 24 hour period. An infusion of 30 grams over the same time period resulted in a maximum ascorbate concentrations of 6.6 mM (130 mg $dl^{-1}$) and an area under the curve 35 mM h. If these infusions were given consecutively over a 2-day period, the exposures would be 152 mM h and 70 mM h, respectively, for the 60 and 30 gram doses. In contrast, 2-day steady state exposure to 10 mM ascorbate is equivalent to an area under the curve of 480 mM $h^{-1}$. Since the total ascorbate exposure obtained in vivo is lower than that necessary for cell killing in SW620 HFST in a 2-day period, we searched for

**Table 1** Effect of 2 day treatment with vitamin C on the percentage of viable, necrotic, and apoptotic cells in SW620 HFST. Percentages were determined using dual staining annexin V and propidium iodide flow cytometry. Results are compared to surviving fractions determined using the SRB assay

| Vitamin C concentration | Flow cytometry analysis | | | Surviving fraction |
|---|---|---|---|---|
| | Viable | Apoptotic | Necrotic | |
| Control | 63.4% | 22.2% | 14.3% | 1.00 |
| 3.74 mM | 50.6% | 29.3% | 20.1% | 0.84 |
| 11.2 mM | 32.8% | 42.9% | 24.4% | 0.63 |
| 33.7 mM | 9.2% | 57.6% | 33.1% | 0.25 |
| 101 mM | 0.9% | 77.6% | 21.6% | 0.00 |

© 2001 Cancer Research Campaign

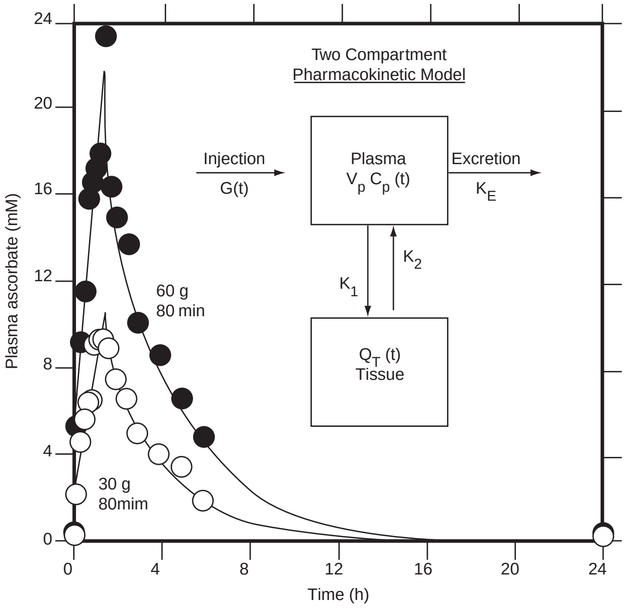

**Figure 2** Vitamin C concentrations in plasma during and after an 80-minute intravenous infusion of 60 (solid circles) or 30 (open circles) grams. The curves represent a least squares fits of the data to the 2 compartment pharmacokinetic model pictured in the figure inset and described in the text, with $K_1$, $K_2$ and $K_E$ values of 0.31 $min^{-1}$, 0.091 $min^{-1}$ and 0.022 $min^{-1}$ for the 60 gram infusion and 0.21 $min^{-1}$, 0.060 $min^{-1}$ and 0.027 $min^{-1}$ for the 30 gram infusion antioxidants that, when used in combination with vitamin C, would reduce the ascorbate $LC_{50}$.

$LC_{50}$ values in SW620 HFST for other reagents, along with $LC_{50}$ data for ascorbate when combined with these agents, are shown in Table 2. The $LC_{50}$ value of phenyl-ascorbate, a lipophilic vitamin C analogue, was roughly 3 times lower than that of the parent compound, suggesting that lipophilic ascorbate analogues may be useful and that lipophilic drug delivery systems may improve ascorbate efficacy. When combined with vitamin C, phenyl ascorbate increased efficacy in an additive fashion. SW620 HFST were very sensitive to vitamin $K_3$. In the combination treatment, vitamin $K_3$ decreased the ascorbate $LC_{50}$ by roughly a factor of 4, though we could not demonstrate synergy. Most interesting were the results with lipoic acid. DL-α-lipoic acid (DL-6,8-thioctic acid) is a lipophilic antioxidant that can be readily obtained commercially in clinical or research reagent grade. Experiments were conducted using both the lipophilic molecule, a form that is likely preferred in vivo, and the sodium salt, a water-soluble form that enables higher concentrations to be used in vitro. Both forms of lipoic acid were toxic to SW620 HFST cells with $LC_{50}$ values of roughly 3 mM. Moreover, both acted synergistically with ascorbate. Dose–response data with ascorbate and the sodium salt of lipoic acid are shown in Figure 3. Using a 10:1 ratio of ascorbate to lipoic acid decreased the ascorbate $LC_{50}$ to 4.5 ± 0.9 mM. This combination killed 25% of SW620 HFST cells at an ascorbate concentration of 1.3 mM. Exposure to ascorbate at this concentration over a 2-day period is comparable to a pharmacokinetic area under the curve of 62 mM h, while exposure to 4.5 mM (the $LC_{50}$) over 2 days is equivalent to an area under the curve of 216 mM h. Our pharmacokinetic data (see above) indicate that exposures of this magnitude can be obtained in human plasma using intravenous vitamin C infusions.

The toxicity of ascorbic acid and lipoic acid (sodium salt) were tested in immortalized cell monolayers of both tumour and normal tissue origin. Results are given in Table 3. With the exception of CCD-18Lu, the fibroblast cell lines were more resistant to these nutrients than the tumour cell lines. The effect of cell proliferation status on sensitivity was tested by using sparse plating densities to allow proliferation and confluent densities to simulate quiescence. The C/S ratio in Table 3 reflects the degree to which non-proliferating cells are more resistant than proliferating cells. Lipoic acid, unlike ascorbate, was equally effective against both proliferating and confluent cell monolayers, suggesting that lipoic acid may target quiescent cells that are resistant to vitamin C.

Because of concerns that vitamin C would protect tumour cells from the effects of chemotherapeutic agents, we conducted experiments in SW620 HFST combining sodium ascorbate with doxorubicin. Results, shown in Figure 4, suggest that the effect of ascorbate on doxorubicin efficacy is concentration dependent. Ascorbate concentrations of 5 mg $dl^{-1}$ (0.25 mM), an amount that might be relevant during oral supplementation, protected SW620 cells from doxorubicin to a small degree, while cytotoxic ascorbate concentrations (500 mg $dl^{-1}$, or 25 mM) increased cell killing. When ascorbate and doxorubicin were combined at a 50 000:1 mass ratio, they had an additive but not synergistic effect, reducing the doxorubicin $LC_{50}$ by nearly 50%. For example, a doxorubicin concentration of 0.02 mM combined with 3 mM vitamin C would kill roughly 25% of SW620 HFST cells in 2 days, while 0.02 mM doxorubicin alone would only kill 5% of the cells.

**Table 2** $LC_{50}$ values for 10-day-old SW620 HFST treated for 2 days with various agents, alone or in combination with sodium ascorbate. In combination treatments, the ratio given is that of ascorbate to the agent listed. $LC_{50}$ values for combination treatments are given in terms of the ascorbate concentration. Errors are given as standard deviations. Additive values are obtained as described in the text. Synergy was determined using Student's *t*-test with standard error estimates from KaleidaGraph

| | | Combination therapy | | |
|---|---|---|---|---|
| **Agent** | **Single agent $LC_{50}$ (mM)** | **Combination mass ratio** | **Actual $LC_{50}$ (mM)** | **Additive $LC_{50}$ (mM)** |
| Phenyl ascorbate | 10.0 ± 2.5 | 10:1 | 24.6 ± 2.0 | 22.9 ± 2.3 |
| Vitamin $K_3$ | 0.19 ± 0.01 | 40:1 | 7.6 ± 0.6 | 8.4 ± 0.8 |
| Lipoic acid (free acid) | 3.0 ± 1.0 | 100:1 | 24.8 ± 3.0[a] | 43.8 ± 4.2 |
| Lipoic acid (sodium salt) | 2.6 ± 0.4 | 10:1 | 4.5 ± 0.6[a] | 9.9 ± 1.1 |
| Doxorubicin | 9.7 (± 1.7) × $10^{-5}$ | 50 000:1 | 7.3 ± 1.0 | 7.5 ± 0.8 |

[a]Different from additive value, with 95% confidence

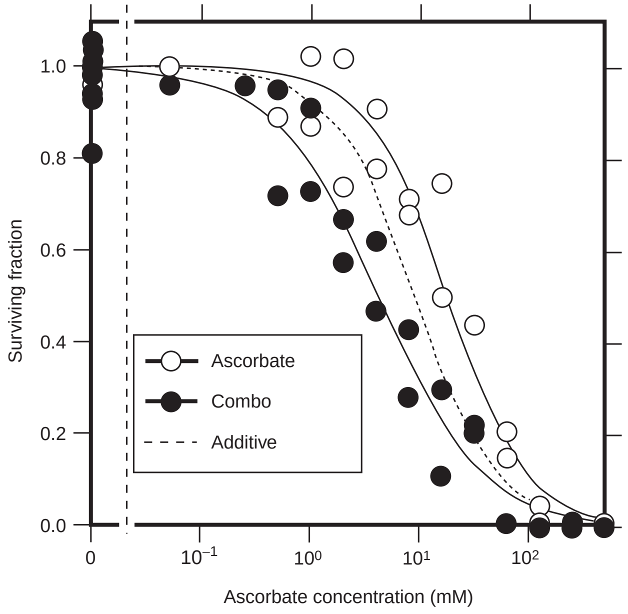

**Figure 3** Dose–response curves for SW620 cells grown 10 days (HFST) inside hollow fibres. Cells were treated for 2 days with vitamin C alone (open circles $LC_{50}$ = 20.4 ± 2.4 mM) or in combination the sodium salt of lipoic acid (closed circles, $LC_{50}$ = 4.3 ± 0.5 mM). The 'additive' curve (no points, dotted line, $LC_{50}$ = 9.8 ± 1.1 mM) represents the product of the surviving fraction for ascorbate alone to that for lipoic acid alone, as described in the text

### DISCUSSION

The results presented above lead to 4 major conclusions. First, sodium ascorbate increases the percentage of apoptotic and necrotic cells in SW620 HFST, though the concentrations necessary for significant cell killing may not be clinically feasible. Second, lipoic acid enhances the anti-tumor efficacy of ascorbate to the point where significant tumour cell killing can occur at concentrations achievable by intravenous infusion. Third, the antioxidants lipoic acid, vitamin $K_3$, and phenyl ascorbate are effective against SW620 HFST in the millimolar range, suggesting their potential use as anticancer agents. Fourth, pro-oxidant concentrations of vitamin C do not negate the cytotoxicity of doxorubicin, though lower ascorbate concentrations may protect tumour cells as well as normal tissues. These results indicate that the use of antioxidants, particularly ascorbate and lipoic acid in combination, as anti-cancer agents warrants further study.

Several mechanisms have been proposed by which vitamin C may be useful in treating cancer, including speculation that it improves immune response, reduces the severity of cachexia, and strengthens extracellular matrix against tumour cell invasion (Cameron et al, 1979; Henson et al, 1991). The ability of ascorbate to kill tumour cells preferentially through hydrogen peroxide generation has been confirmed in several in vitro studies (Bram et al, 1980; Leung et al, 1993) (Benade et al, 1969; Riordan et al, 1995). Rodent studies indicate that ascorbate supplementation can inhibit tumour growth in vivo (Tsao et al, 1988; Varga and Airoldi, 1983). The present study supports a cytotoxic effect of ascorbate with one major caveat: cells in SW620 HFST were much more resistant to ascorbate than SW620 cell monolayers. This may relate to the presence of quiescent cells in SW620 HFST, since monolayer data indicate that ascorbate is less effective in treating non-proliferating cells. Cellular transport and intra-tumor diffusion may also be an issue, since the $LC_{50}$ of phenyl ascorbate, a lipophilic analogue of vitamin C, was roughly 3 times lower than that of sodium ascorbate.

The high concentrations of ascorbate needed for significant cell killing led to our interest in combining it with other antioxidants. Previous reports suggest a synergistic relationship between vitamins C and $K_3$, 2 hydrogen peroxide generators, using a 100:1 molar ratio against tumour cell monolayers (Noto et al, 1989). We failed to find a synergistic relationship using a 40:1 mass ratio (a roughly 55:1 molar ratio) in SW620 HFST. Proliferative and microenvironment heterogeneity in HFST may reduce the combined effect of the 2 agents. The use of lipoic acid in the treatment of cancer has not, to our knowledge, been previously studied. Lipoic acid is a lipophilic antioxidant that, among other things, inhibits hydrogen peroxide generation by ascorbate in erythrocytes (Ou et al, 1995). We were thus surprised to find that not only was lipoic acid toxic to tumour cells at millimolar concentrations, but that it enhanced the cytotoxicity of ascorbate in a synergistic fashion. The reason for this synergy is unknown. Chemically, it may relate to the ability of these nutrients to modify cellular oxidation-reduction status in a manner that enhances $H_2O_2$ toxicity (Jonas et al, 1989) or to possible ascorbate recycling by lipoic acid in redox reactions. Biologically, the effectiveness of lipoic acid in confluent cell monolayers suggests that it may aid in killing non-proliferating SW620 HFST cells that are relatively resistant to ascorbate. We do not know to what extent the effects of lipoic acid

**Table 3** $LC_{50}$ values for 7 cell lines treated for 3 days with either sodium ascorbate or the sodium salt of lipoic acid. The C/S ratio represents the ratio of the $LC_{50}$ for confluent cells to that for sparse (proliferating) cells. Cell line origins are given in the text. Errors given as standard deviations

| Cell line | Ascorbic acid (sodium salt) $LC_{50}$ (mM) Confluent | Sparse | C/S ratio | Cell line | Lipoic acid (sodium salt) $LC_{50}$ (mM) Confluent | Sparse | C/S ratio |
|---|---|---|---|---|---|---|---|
| CCD-18Co | no data | 4.5 ± 0.9 | no data | CCD-18Co | no data | 7.3 ± 1.5 | no data |
| CCD-18Lu | no data | 1.3 ± 0.9 | no data | CCD-18Lu | no data | 2.2 ± 0.6 | no data |
| CCD-25Sk | no data | 7.3 ± 4.3 | no data | CCD-25Sk | no data | 16.4 ± 2.6 | no data |
| SW620 | 5.6 ± 0.7 | 1.8 ± 0.3 | 3.1 | SW620 | 2.8 ± 0.5 | 3.5 ± 0.4 | 0.8 |
| SK-MEL | 2.7 ± 0.5 | 1.7 ± 0.3 | 1.6 | SK-MEL | 1.1 ± 0.1 | 2.0 ± 0.3 | 0.6 |
| Mia PaCa | 5.0 ± 0.7 | 1.3 ± 0.2 | 3.8 | Mia PaCa | 2.7 ± 0.6 | 2.6 ± 0.6 | 1.0 |
| MCF-7 | 1.4 ± 8.5 | 0.6 ± 0.1 | 2.3 | MCF-7 | 2.6 ± 0.3 | 3.3 ± 1.3 | 0.8 |

 *© 2001 Cancer Research Campaign*

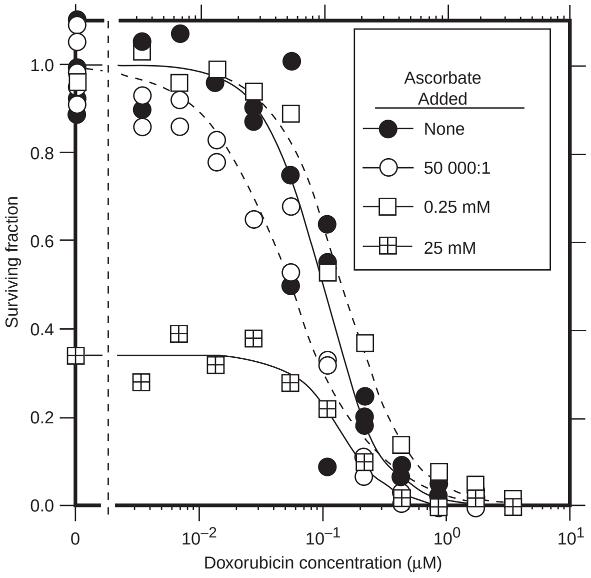

**Figure 4** Dose–response curves for SW620 cells grown 10 days (HFST) inside hollow fibres. Cells were treated for 2 days with doxorubicin alone (closed circles, $LC_{50}$ = 0.097 ± 0.017 μM), with 50 000 grams ascorbate per gram doxorubicin (open circles, $LC_{50}$ = 0.054 ± 0.014 μM), with a fixed ascorbate concentration of 0.25 mM (open boxes, $LC_{50}$ = 0.142 ± 0.010 μM), and with a fixed ascorbate concentration of 25 mM (crossed squares, surviving fraction of 0.34 without doxorubicin)

and ascorbate on cells in SW620 HFST carry over to other solid tumour models with different cell types and potentially different microenvironments. However, the cytotoxicity of lipoic acid and its effect on confluent monolayers were evident in all 4 of the tumour cell lines we tested, and these cells had similar lipoic acid $LC_{50}$ values.

One key in assessing the value of ascorbate anti-cancer agent is to compare the concentrations required for tumour cell killing with those that can be safely achieved in the clinic. In the case of ascorbate, we found that intravenous infusions of 30 to 60 grams could provide plasma ascorbate exposures (area under the curve) that would be effective against SW620 HFST if lipoic acid were also present. The HFST model does not allow for long-term studies, due to limited space for expansion of tumour cells. Increased treatment times available in vivo should reduce the concentration required for efficacy. Given this suggestion of feasibility, 2 key issues arise: the relationship between plasma and tumour ascorbate concentrations, and the safety of intravenous infusions on the order of 50 grams. 2 lines of evidence suggest that tumour ascorbate levels may be higher than those in plasma. First, vitamin C concentrations in tumours are, on the average, 3 times higher than those in surrounding normal tissues (Langemann et al, 1989), presumably because tumour cells use membrane glucose transporters to internalize ascorbate (Agus et al, 1999). Secondly, tissue vitamin C concentrations in guinea pigs and humans exceed those in plasma by as much as 2 orders of magnitude (Hornig, 1975). Vitamin C may have a favourable therapeutic index, despite the high concentrations needed for cytotoxicity, if it accumulates in tumours and lacks systemic toxicity. Measurements of tumour ascorbate levels in animals given high doses of ascorbate and lipoic acid are needed to definitively resolve this issue.

In regards to safety, some of us (NHR, HDR) have been giving infusions of this magnitude to patients for years (Jackson et al, 1995; Riordan et al, 1998) without ill effect. While vitamin C has shown no major side effects in published clinical trials, the doses used in these trials have not exceeded 10 grams per day (Cameron and Campbell, 1974; Creagan et al, 1979). Studies at higher doses are needed. A recent study (manuscript in preparation, Casciari JJ, Tempero MA, Riordan NH, Rodrigues G, Taylor P, Jackson JA et al) at the University of Nebraska indicates that blood count and chemistry parameters are relatively stable in terminal cancer patients given continuous infusions of up to 50 grams per day for up to 8 weeks. We are not at this time aware of any clinical trials testing the safety of lipoic acid at high doses.

The accumulation of ascorbate in tumours has raised fears among some that it may protect tumour cells from oxidative damage associated with traditional therapeutic modalities (Raloff, 2000). However, studies in cell culture suggest that vitamin C can enhance the efficacy of chemotherapy (Kurbacher et al, 1996) and in vivo data suggest the same for radiation (Taper et al, 1996). In fact, the data by Kurbacher and co-workers testing doxorubicin efficacy in cell monolayers suggest synergy with vitamin C at 1 μM or 0.1 mM. Our data with SW620 HFST suggest that ascorbate protects tumour cells from doxorubicin at low concentrations (0.25 mM). This discrepancy may relate to the different models used. Drug concentrations in monolayer experiments are straightforward, while HFST can contain significant drug concentration gradients (Casciari et al, 1994). It may be that the 'protective' effects of ascorbate observed in HFST occur in the inner regions, where doxorubicin and perhaps ascorbate uptake is lower than at the proliferating rim. Also, the effects may be cell line dependent, as Kurbacher and co-workers used breast cell lines while we used a colon cell line. Our data suggest that pro-oxidant ascorbate concentrations relevant during intravenous infusions above 10 grams will add to rather than negate the tumour cell killing of doxorubicin. To properly resolve this issue, though, we must learn more about the actual ascorbate and doxorubicin concentrations in solid tumours during chemotherapy and vitamin supplementation. Moreover, we must balance the potential negative effects of ascorbate on doxorubicin efficacy against potential benefits such as alleviation of vitamin C depletion, restoration of immune system function and protection of normal tissues from therapeutic side effects.

### REFERENCE

Agus DB, Vera JC and Golde DW (1999) Stromal cell oxidation: a mechanism by which tumors obtain vitamin C. *Cancer Res* **59**: 4555–4558

Benade L, Howard T and Burk D (1969) Synergistic killing of Ehrlich ascites carcinoma cells by ascorbate and 3-amino-1,2,4,-triazole. *Oncology* **23**(1): 33–43

Bram S, Froussard P, Guichard M, Jasmin C, Augery Y, Sinoussi-Barre F and Wray W (1980) Vitamin C preferential toxicity for malignant melanoma cells. *Nature* **284**(5757): 629–631

Cameron E and Campbell A (1974) The orthomolecular treatment of cancer. II. Clinical trial of high-dose ascorbic acid supplements in advanced human cancer. *Chem Biol Interact* **9**(4): 285–315

Cameron E and Pauling L (1976) Supplemental ascorbate in the supportive treatment of cancer: Prolongation of survival times in terminal human cancer. *Proc Natl Acad Sci USA* **73**(10): 3685–3689

Cameron E, Pauling L and Leibovitz B (1979) Ascorbic acid and cancer: a review. *Cancer Res* **39**(3): 663–681

Casciari JJ, Hollingshead MG, Alley MC, Mayo JG, Malspeis L, Miyauchi S, Grever MR and Weinstein JN (1994) Growth and chemotherapeutic response of cells in a hollow fiber in vitro solid tumor model. *J Natl Cancer Inst* **86**: 1846–1852

Collins JM (1996) Pharmacokinetics and clinical monitoring. In *Cancer Chemotherapy and Biotherapy*, Chabner BA and Longo DL (eds) pp 17–30. Lippincott-Raven Publishers: Philadelphia

© 2001 Cancer Research Campaign

Creagan ET, Moertel CG, O'Fallon JR, Schutt AJ, O'Connell MJ, Rubin J and Frytak S (1979) Failure of high-dose vitamin C (ascorbic acid) therapy to benefit patients with advanced cancer. A controlled trial. *N Engl J Med* **301**(13): 687–690

Fujita K, Shinpo K, Yamada K, Sato T, Niimi H, Shamoto M, Nagatsu T, Takeuchi T and Umezawa H (1982) Reduction of adriamycin toxicity by ascorbate in mice and guinea pigs. *Cancer Res* **42**(1): 309–316

Goldschmidt MC (1991) Reduced bactericidal activity in neutrophils from scorbutic animals and the effect of ascorbic acid on these target bacteria in vivo and in vitro. *Am J Clin Nutr* **54**(6 Suppl): 1214S–1220S

Henry RJ, Cannon DC and Winkleman JW (1974) Determination of Ascorbic Acid. In: *Clinical Chemistry: Principles and Techniques* pp 1393–1398. Harper & Row: New York

Henson DE, Block G and Levine M (1991) Ascorbic acid: biologic functions and relation to cancer. *J Natl Cancer Inst* **83**(8): 547–550

Hornig D (1975) Distribution of ascorbic acid, metabolites and analogues in man and animals. *Ann N Y Acad Sci* **258**: 103–118

Jackson JA, Riordan HD, Hunninghauke RE and Riordan N (1995) High dose intravenous vitamin C and long time survival of a patient with cancer of the head of the pancreas. *J Ortho Med* **10**: 87–88

Jonas SK, Riley PA and Willson RL (1989) Hydrogen peroxide cytotoxicity: low-temperature enhancement by ascorbate or reduced lipoate. *Biochem J* **265**: 651–655

Koch CJ and Biaglow JE (1978) Toxicity, radiation sensitivity modification, and metabolic effects of dehydroascorbate and ascorbate in mammalian cells. *J Cell Physiol* **94**(3): 299–306

Kurbacher CM, Wagner U, Kolster B, Andreotti PE, Krebs D and Bruckner HW (1996) Ascorbic acid (vitamin C) improves the antineoplastic activity of doxorubicin, cisplatin, and paclitaxel in human breast carcinoma cells in vitro. *Cancer Lett* **103**(2): 183–189

Langemann H, Torhorst J, Kabiersch A, Krenger W and Honegger CC (1989) Quantititve determination of water and lipid soluble antioxidants in neoplastic and non-neoplastic human breast tissue. *Int. J. Cancer* **43**: 1169–1173

Leung PY, Miyashita K, Young M and Tsao CS (1993) Cytotoxic effect of ascorbate and its derivatives on cultured malignant and nonmalignant cell lines. *Anticancer Res* **13**(2): 475–480

Moertel CG, Fleming TR, Creagan ET, Rubin J, O'Connell MJ and Ames MM (1985) High-dose vitamin C versus placebo in the treatment of patients with advanced cancer who have had no prior chemotherapy. A randomized double-blind comparison. *N Engl J Med* **312**(3): 137–141

Murata A, Morishige F and Yamaguchi H (1982) Prolongation of survival times of terminal cancer patients by administration of large doses of ascorbate. *Int J Vitam Nutr Res Suppl* **23**: 103–113

Noto V, Taper HS, Jiang YH, Janssens J, Bonte J and De Loecker W (1989) Effects of sodium ascorbate (vitamin C) and 2-methyl-1,4-naphthoquinone (vitamin K3) treatment on human tumor cell growth in vitro. I. Synergism of combined vitamin C and K3 action. *Cancer* **63**(5): 901–906

Okunieff P and Suit HD (1987) Toxicity, radiation sensitivity modification, and combined drug effects of ascorbic acid with misonidazole in vivo on FSaII murine fibrosarcomas. *J Natl Cancer Inst* **79**(2): 377–381

Ou P, Tritschler HJ and Wolff SP (1995) Thioctic (lipoic) acid: a therapeutic metal-chelating antioxidant? *Biochemical Pharmacology* **50**: 123–126

Penn ND, Purkins L, Kelleher J, Heatley RV, Mascie-Taylor BH and Belfield PW (1991) The effect of dietary supplementation with vitamins A, C and E on cell-mediated immune function in elderly long-stay patients: a randomized controlled trial. *Age Ageing* **20**(3): 169–174

Raloff J (2000) Antioxidants may help cancers thrive. *Science News* **157**: 5

Riordan HD, Jackson JA, Riordan NH and Schultz M (1998) High-dose intravenous vitamin C in the treatment of a patient with renal cell carcinoma of the kidney. *J Ortho Med* **13**: 72–73

Riordan HR, Jackson JA and Schultz M (1990) Case study: high-dose intravenous vitamin C in the treatment of a patient with adenocarcinoma of the kidney. *J Ortho Med* **5**: 5–7

Riordan NH, Riordan HD, Meng X, Li Y and Jackson JA (1995) Intravenous ascorbate as a tumor cytotoxic chemotherapeutic agent. *Med Hypotheses* **44**(3): 207–213

Taper HS, Keyeux A and Roberfroid M (1996) Potentiation of radiotherapy by nontoxic pretreatment with combined vitamins C and K3 in mice bearing solid transplantable tumor. *Anticancer Res* **16**(1): 499–503

Tsao CS, Dunham WB and Leung PY (1988) In vivo antineoplastic activity of ascorbic acid for human mammary tumor. *In Vivo* **2**(2): 147–150

Varga JM and Airoldi L (1983) Inhibition of transplantable melanoma tumor development in mice by prophylactic administration of Ca-ascorbate. *Life Sci* **32**(14): 1559–1564

 *© 2001 Cancer Research Campaign*
